# How did *Bursaphelenchus* nematodes acquire a specific relationship with their beetle vectors, *Monochamus*?

**DOI:** 10.3389/fphys.2023.1209695

**Published:** 2023-07-31

**Authors:** Haru Kirino, Noritoshi Maehara, Ryoji Shinya

**Affiliations:** ^1^ School of Agriculture, Meiji University, Kawasaki, Japan; ^2^ Department of Forest Entomology, Forestry and Forest Products Research Institute, Tsukuba, Japan

**Keywords:** dauer, *Bursaphelenchus*, beetle vector, evolution, dauer-inducing

## Abstract

For insect-borne pathogens, phoretic ability is important not only to spread more widely and efficiently but also to evolve virulence. *Bursaphelenchus xylophilus*, the causal agent of pine wilt disease, is transmitted by the cerambycid beetle *Monochamus alternatus*, which is associated with pine tree host. Their specific phoretic ability to appropriate vectors depending on their life cycle is critical for efficient transfer to the correct host and is expected to enhance virulence. We evaluated how *B*. *xylophilus* acquired a specific relationship with *M*. *alternatus* with a focus on *Bursaphelenchus okinawaensis*, a close relative of *B*. *xylophilus* that has evolved a relationship with a cerambycid beetle vector. *Bursaphelenchus okinawaensis* has a single dispersal stage (dauer) larva (third-stage dispersal [DIII] larva), whereas *B*. *xylophilus* has two distinct dispersal stages (DIII and fourth-stage dispersal [DIV] larva). Also, the dauer formation in *B. okinawaensis* is not completely dependent on its beetle vector, whereas DIV larvae of *B*. *xylophilus* are induced by volatile from the beetle vector. We investigated the induction conditions of dauer larvae in *B*. *okinawaensis* and compared to with *B*. *xylophilus*. The dauer percentages of *B*. *okinawaensis* significantly increased when the nematode population on the plate increased or when we propagated the nematodes with a crude extract of cultured nematodes, which likely contained dauer-inducing pheromones. In addition, dauer formation tended to be enhanced by the crude extract at high temperatures. Furthermore, when we propagated the nematodes with *M*. *alternatus* pupae until the beetles eclosed, *B*. *okinawaensis* significantly developed into dauer larvae. However, only 1.3% of dauer larvae were successfully transferred to *M*. *alternatus*, the rate lower than that of *B*. *xylophilus*. DIII and DIV of *B*. *xylophilus* were induced by increasing the nematode population and the presence of the beetle vector, respectively. These results suggest that *B. okinawaensis* has acquired specificity for the cerambycid beetle through dauer formation, which is efficiently induced in the presence of the beetle, and the DIV larval stage, exclusive to the xylophilus group, may be crucial for high transfer ability to the beetle vector.

## Introduction

The virulence of pathogens is a trade-off with their transmissibility (Ebert and Herre, 1996; Ebert and Bull, 2003). For example, although high virulence improves the success rate of infection, such pathogens kill their hosts quickly and thus have fewer opportunities to encounter new hosts. By contrast, pathogens with low virulence are expected to increase the available time for transmission because they can survive in the host longer than those with high virulence. According to this trade-off model, pathogens can increase their virulence (via evolution by natural selection) when they can disperse readily from even immobilized or dead hosts by the infection (Ewald, 2004). That is, all pathogens are expected to have improved their transmissibility before gaining virulence, and higher virulence needs higher transmissibility. Some pathogens have evolved to use animal vectors to effectively transmit to new hosts. Insects transmit various species of pathogen. These insect-borne pathogens are more lethal on a per-infection basis than directly transmitted pathogens (Ewald, 1983). Therefore, phoretic ability of pathogens to attach to vector insects can promote not only the spread of pathogens but also their virulence.

*Bursaphelenchus xylophilus* (Steiner and Buhrer, 1934) Nickle, 1970 (Nematoda, Aphelenchoididae) is the causal pathogen of pine wilt disease, one of the most serious forest diseases globally, and most strains of *B*. *xylophilus* have both high virulence and high transmissibility via insect vectors (Kiyohara and Tokushige, 1971; Mamiya and Enda, 1972; Morimoto and Iwasaki, 1972). After nematode infection, host pine trees show sequential and excessive hypersensitivity responses, leading to the death of susceptible pine trees (Myers, 1988; Futai, 2003; Hirao et al., 2012). Although *B*. *xylophilus* is thought to use one host for a very short time, it can disperse efficiently to new hosts because a large number of nematodes are vectored by the specific cerambycid beetle *Monochamus alternatus* Hope, 1843 (Coleoptera: Cerambycidae), which emerges from dead pine trees and disperses to feed on new, living pine trees (Morimoto and Iwasaki, 1972; Togashi, 1985). This *B*. *xylophilus*–*M*. *alternatus* relationship fits well the virulence trade-off model. Wild strains of *B*. *xylophilus* of high virulence tend to have a high dispersal ability, whereas low-virulence strains tend to have low dispersal abilities (Togashi and Jikumaru, 2007). Therefore, establishment of a specific one-to-one relationship between *B*. *xylophilus* and *M. alternatus* is critical for not only efficient transfer to the correct host but also promotion of their virulence.

In this study, we evaluated how *B*. *xylophilus* acquired this specific relationship with *M*. *alternatus*. *Bursaphelenchus xylophilus* has two developmental forms, the normal propagative form and the dispersal form (the dauer) (Kanzaki and Giblin-Davis, 2018). In the xylophilus group of *Bursaphelenhus* (Braasch, 2001; Kanzaki and Futai, 2002; Kanzaki and Giblin-Davis, 2018), nematodes develop into third-stage dispersal (DIII) larvae from the propagative form when the population density increases (Tanaka et al., 2017). The DIII larvae molt to fourth-stage dispersal (DIV) larva, which is analogous to the dauer stage in *Caenorhabditis elegans* (Maupas, 1899) Dougherty, 1953 (Nematoda, Rhabditidae) and the insect-phoretic stage, when late pupae and callow adults of cerambycid beetles (*Monochamus* spp., *Acalolepta* spp., and *Psacothea hilaris* Pascoe, 1857) are present in a pupal chamber (Morimoto and Iwasaki, 1973; Maehara and Futai, 1996; 2001; Necibi and Linit, 1998; Maehara et al., 2020). The formation of the DIII larva of *B*. *xylophilus* is promoted by crude extracts of cultured nematodes (Tanaka et al., 2017), suggesting that these larvae are induced by the water-soluble pheromone ascaroside, as is the free-living nematode *C. elegans* (Golden and Riddle, 1982; Jeong et al., 2005; Butcher et al., 2007; Butcher et al., 2008). Zhao et al. (2013) reported that DIV larvae of *B*. *xylophilus* were induced by volatile C16 and C18 fatty acid ethyl esters, which are abundant on the body surface of the beetle vector. However, it is unclear how the dauer-inducing mechanisms evolved, resulting in a close relationship with a particular vector. Furthermore, although the presence of two distinct dauer stages is unique to the ‘xylophilus’ group, its evolutionary significance is unknown. The answers to these questions will provide insight into the evolutionary process of pathogens not only of pine wilt disease but also other vector-borne infectious diseases.

We investigated the dauer-inducing conditions of *Bursaphelenchus okinawaensis* Kanzaki, Maehara, Aikawa, and Togashi, 2008 (Nematoda, Aphelenchoididae), which has a single dauer stage (DIII) larva, and compared them to *B*. *xylophilus*, which has two distinct dauer stages (Maehara and Futai, 2001; Zhao et al., 2013). Although *B*. *okinawaensis* is a close relative of *B*. *xylophilus*, and its natural vector is *Monochamus* cerambycid beetle (*M. maruokai* Hayashi, 1962 (Coleoptera: Cerambycidae)), the DIII stage of *B*. *okinawaensis* can be induced on solid fungal culture medium without its beetle vector (Kanzaki et al., 2008). This suggests that the dauer formation in *B. okinawaensis* is not completely dependent on its beetle vector and may be in the process of acquiring a close relationship with its vector. In this study, 1) we performed artificial dauer induction of *B*. *okinawaensis* using crude extracts of cultured nematodes, which likely contain dauer-inducing pheromone-like substances. 2) We investigated the effect of temperature on dauer formation because the dauer formation of some nematode species, including *C*. *elegans*, increases at high temperatures (Golden and Riddle, 1984). 3) We also examined if dauer larvae of *B*. *okinawaensis* could be induced by co-cultivation with *M*. *alternatus*, which is of the same genus as its natural vector, *Monochamus maruokai*. Our results provide insight into the evolution of the relationship between *B*. *xylophilus* and its beetle vector.

## Materials and methods

### Nematode strains and culturing

*Bursaphelenchus okinawaensis* (SH1 isolate) was propagated on the fungus *Botrytis cinerea* Pers. Fr. 1794 (Helotiales: Sclerotiniaceae) at 25°C on malt extract agar (1.5% malt extract [Becton Dickinson] and 4% agar [Rikaken]) containing 100 µg/mL chloramphenicol (Fujifilm) in 90 mm Petri dishes (Shinya et al., 2014).

### Beetle culturing

*Monochamus alternatus* was collected from logs of *Pinus thunbergia* killed by pine wilt disease at the Forest Tree Breeding Station, the Iwate Prefectural Forestry Technology Center, Oshu, Iwate, Japan. The beetles were propagated as described previously (Maehara and Kanzaki, 2016; Maehara et al., 2020). *Monochamus alternatus* was oviposited and hatched on *P*. *densiflora* Sieb. and Zucc, and the larvae were cultivated with approximately 20 g artificial diet (8 g current and 1-year-old needles of *P*. *densiflora* dried at 70°C for 1 day and milled into powder), 26.8 g artificial silkworm diet (Silkmate 2M powder, Nosan), 3.2 g dried yeast (EBIOS, Asahi Food & Healthcare), and 62 mL distilled water) in a 50 mL Erlenmeyer flask. The larvae were reared at 25°C in the dark for 3–4 months, and mature larvae were incubated at 10°C in the dark for 14–15 months. Thereafter, mature larvae were washed with distilled water, dipped for 5 s in 70% ethanol, and washed again in sterilized distilled water (SW). Larvae were placed on wet filter paper with 1 mL SW at 25°C for about 2 weeks in the dark to develop into pupae.

### Extraction of dauer-inducing pheromone-like substances

Nematodes propagated with *B*. *cinerea* were collected using the Baermann funnel technique for 3 h, washed three times with SW, and re-suspended in SW (10 worms/μL). The suspension was shaken at 120 rpm for 48 h at 25°C, and passed through 0.45 μm filter paper (AS ONE Corporation) to remove nematodes. The filtered solution was concentrated by freeze-drying (PFR-1000 and FDS-1000; Tokyo Rikakikai), and the dried materials were dissolved in SW and adjusted to 300 worms/μL. The solution was passed through a 0.2 μm membrane filter (ADVANTEC) and used as the crude nematode culture extract.

### Induction of dauer formation by population density

*Botrytis cinerea* was inoculated on 4% plain (water) agar containing 100 µg/mL chloramphenicol in 40 mm Petri dishes, and incubated for 1 week at 25°C. Fifteen young adults of *B*. *okinawaensis* were incubated on the *B*. *cinerea* plates, and were propagated at 25°C for 5, 10, and 15 days. The dishes were wrapped in Parafilm M^®^ (Bemis Flexible Packaging). Next, nematodes were collected using the Baermann funnel technique overnight. The numbers of nematodes (except second-stage larvae) and dauers were determined for each sample using a light microscope (SMZ1500; Nikon), and the percentage of dauers was calculated. Each treatment was repeated six times.

### Induction of dauer formation on pheromone plates

Nematode crude culture extract (200 μL) was spread on 4% plain (water) agar containing 100 µg/mL chloramphenicol in 40 mm Petri dishes. In the control group, the same volume of SW was used instead of crude extract. The plates were dried and used to culture *B*. *cinerea* for 2 days at 25°C. Fifteen young adults of *B*. *okinawaensis* were incubated on the *B*. *cinerea* plates. Each plate was stored at only 25°C for 5 days to examine the pheromone effects, and at 20°C, 25°C, or 30°C for 5 days to examine the temperature effects. The dishes were wrapped in Parafilm M^®^. The nematodes were counted and the percentage of dauers was calculated as above. Each treatment was repeated six times.

### Induction of dauer formation by co-incubation with *M*. *alternatus*

Agar disks (7 mm diameter) of *N. viridescens* (Hypocreales: Nectriaceae) were placed on malt extract agar (1.5% malt extract and 5% agar) containing 100 µg/mL chloramphenicol in 90 mm Petri dishes, and incubated at 25°C for 5 days. Five hundred mixed-stage *B*. *okinawaensis* were incubated on *Nectria viridescens* plates at 25°C for >5 days. Pupae of *M*. *alternatus* 7 days after pupation were placed on the plate and incubated at 25°C for >10 days. In the control group, nematodes were not co-incubated with pupae. The dishes were wrapped in Parafilm M^®^ (Bemis Flexible Packaging). Fifteen days after nematode inoculation, each beetle was rinsed with distilled water, and ground for 10 s using a blender in 40 mL distilled water (Maehara and Kanzaki, 2016; Maehara et al., 2020). Nematodes were collected from the surfaces of beetles, ground beetles, and agar plates using the Baermann funnel technique overnight. Only dauers collected from ground beetles were counted as “dauers on beetles.” We used only plates whose adult beetles eclosed within 14 days after nematode inoculation. The number of nematodes was counted and the percentage of dauers was calculated as above. Each treatment was repeated 10 times.

## Results

### Population density and crude extracts of cultured nematodes induce dauer formation

**FIGURE 1:**
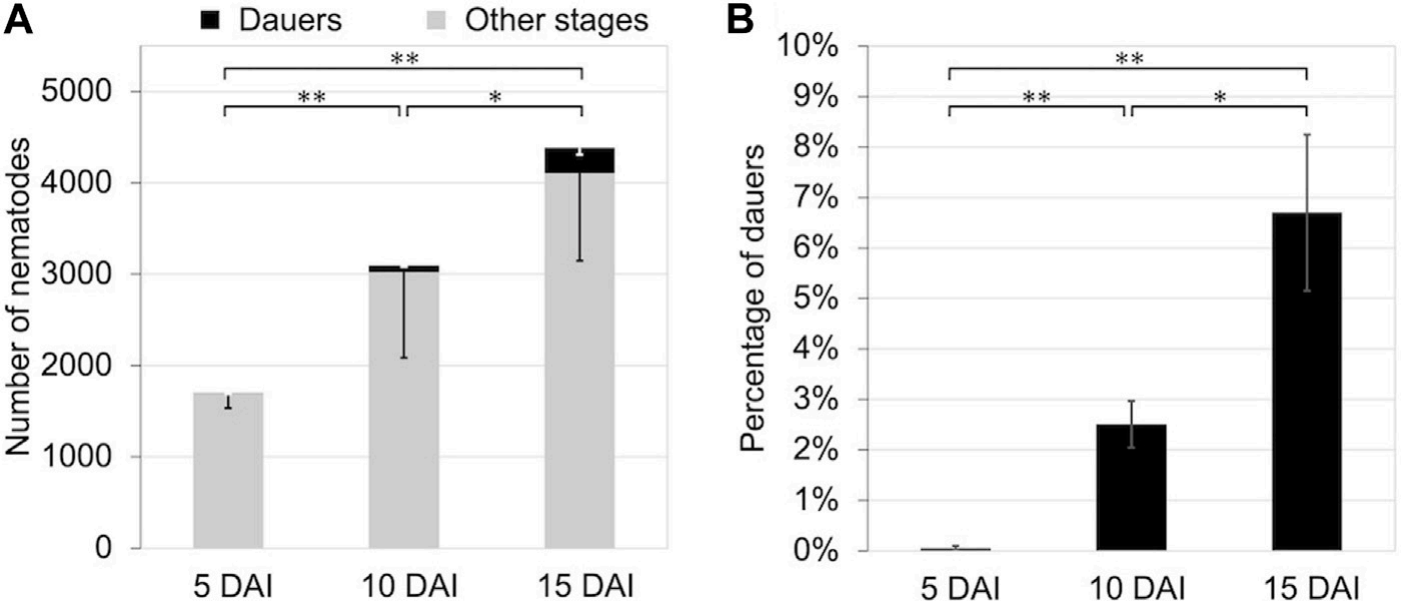
Number and percentage of dauers of *Bursaphelenchus okinawaensis* at 25°C at 5, 10, and 15 days after inoculation (DAI). **(A)** Numbers of dauers and other stages of the nematode (L3–adult). **(B)** Percentage of dauers except second-stage larvae calculated from the data shown in Figure 1A. Bars and error bars are averages and standard errors, respectively. The *t*-test was used to compare dauer numbers and percentages among the 5, 10, and 15 DAI (**p < 0.01; *p < 0.05).

We propagated *B*. *okinawaenis* on 40 mm plates at 25°C and investigated the relationship between the total number of nematodes and the percentage of dauers at 5, 10, and 15 days after inoculation (DAI). The total number of nematodes propagated increased, and the percentages significantly increased between 5 DAI and 15 DAI (*t*-test: t = 4.30, df = 5, *p* < 0.01; Figure 1). Similarly, there were significant differences in the percentages of dauers between 5 DAI and 10 DAI and between 10 DAI and 15 DAI, (*t*-test: t = 5.27, df = 5, *p* < 0.01; t = 2.60, df = 6, *p* < 0.05).

**FIGURE 2:**
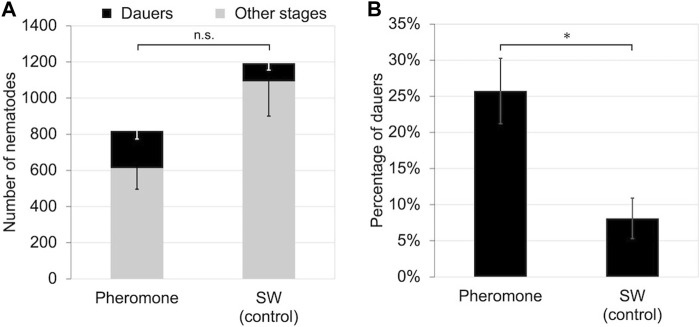
Number and percentage of dauers of *B. okinawaensis* incubated at 25°C with or without crude nematode culture extract after 5 days of incubation. **(A)** Numbers of dauers and other stages of the nematode (L3–adult). **(B)** Percentage of dauers except second-stage larvae calculated from the data shown in Figure 2A. *B*. *okinawaensis* was incubated with crude nematode culture extract or sterilized distilled water (SW) as the control. Bars and error bars are averages and standard errors, respectively. The *t*-test was used to compare dauer numbers and percentages according to the presence of crude extract (*p < 0.05; n.s. *p* > 0.05).

To determine if *B*. *okinawaensis* senses population density via a water-soluble substance, we propagated the nematode on 40 mm plates with crude extracts of cultured nematodes at 25°C. Although the number of dauers induced did not differ significantly between the pheromone-treatment and control groups (*t*-test: t = 1.82, df = 10, *p* > 0.05), the rate of dauer formation was significantly higher (*t*-test: t = 3.31, df = 8, *p* < 0.05) in the pheromone-treatment group (Figure 2).

### High temperatures induce dauer formation

**FIGURE 3:**
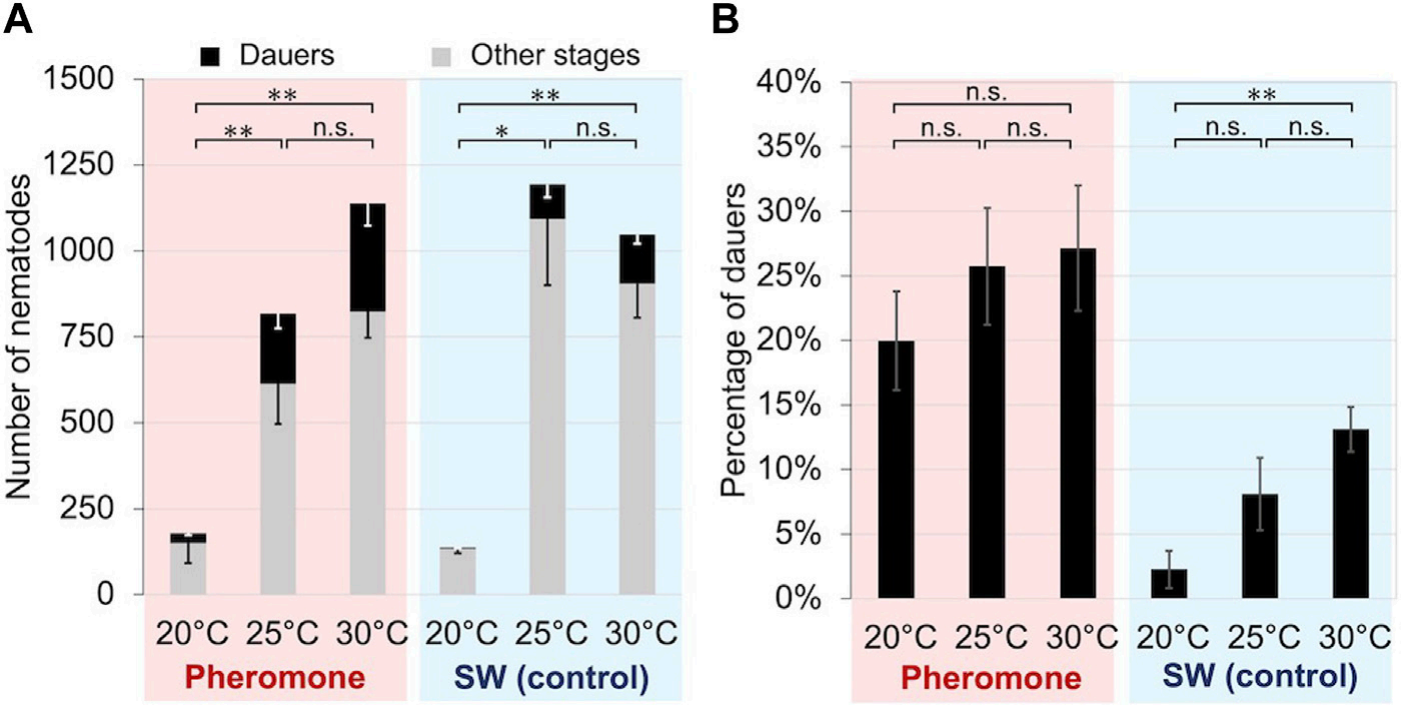
Propagation and dauer formation of *B. okinawaensis* incubated at different temperatures with or without crude nematode culture extract. **(A)** Numbers of dauers and other stages of the nematode (L3–adult). **(B)** Percentage of dauers except second-stage larvae calculated from the data shown in Figure 3A. *B. okinawaensis* was incubated at 20°C, 25°C, or 30°C with crude nematode culture extract or with SW as the control. Bars and error bars are averages and standard errors, respectively. The *t*-test was used to compare dauer numbers and percentages among the different temperature treatments (**p < 0.01; *p < 0.05; n.s. *p* > 0.05).

We propagated the nematode with crude extracts of cultured nematodes at 20°C, 25°C, or 30°C for 5 days. Significantly more dauer larvae were induced at 25°C and 30°C than at 20°C in both the crude extract treatment group (*t*-test: t = 4.01, df = 5, *p* < 0.01; t = 4.40, df = 5, *p* < 0.01) and the control group (*t*-test: = 2.55, df = 5, *p* < 0.05; t = 5.33, df = 5, *p* < 0.01), although the total number of *B*. *okinawaensis* propagated was small at 20°C (Figure 3). The percentage of dauer larvae induced tended to increase with increasing temperature, although there were no significant differences among the three temperatures except between 20°C and 30°C in the control group (*t*-test: t = 4.80, df = 10, *p* < 0.01).

### Co-incubation with *M*. *alternatus* induces dauer formation

**FIGURE 4:**
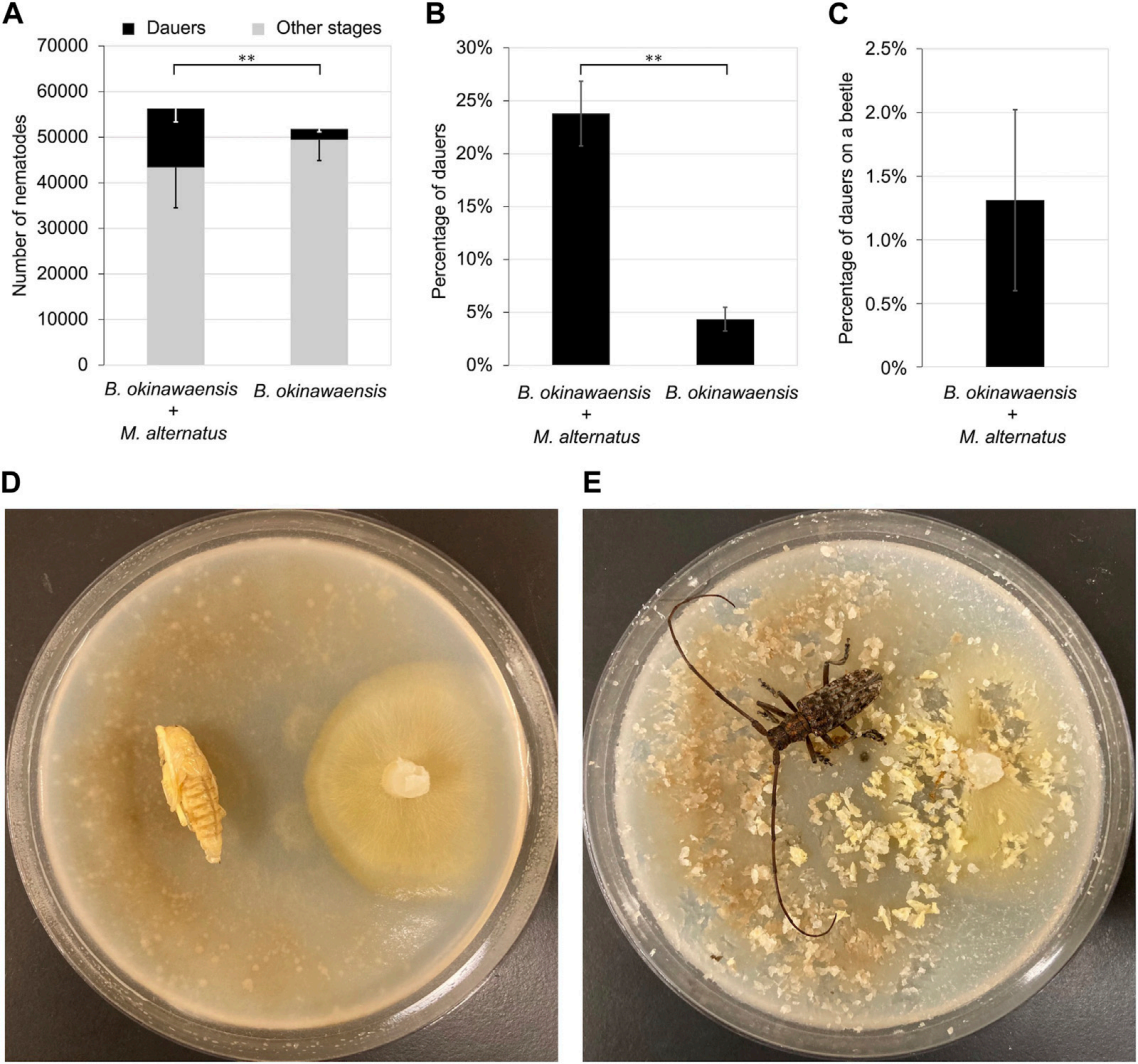
Propagation and dauer formation of *B. okinawaensis* incubated with *Monochamus alternatus*. **(A)** Total numbers of dauers and other stages of the nematode (L3–adult). **(B)** Percentage of dauers except second-stage larvae calculated from the data shown in Figure 4A. **(C)** Percentage of dauers on beetles per total dauer. **(D,E)** *B. okinawaensis*–*M*. *alternatus* co-incubation plate; **(D)** before and **(E)** after adult eclosion. *B. okinawaensis* was incubated with pupae of *M*. *alternatus* until eclosion. Bars and error bars are averages and standard errors, respectively. The *t*-test was used to compare dauer numbers and percentages according to the presence of pupae of *M*. *alternatus* (**p < 0.01).

We incubated the nematodes with the pupae of *M*. *alternatus* until adults eclosed at 25°C. There were no significant differences in the total number of nematodes between the co-incubation group with *M*. *alternatus* and the control group (Figure 4). By contrast, the number of dauers and the rate of dauer formation in the co-incubation group were significantly higher than in the control group (*t*-test: t = 3.53, df = 10, *p* < 0.01; t = 6.70, df = 12, *p* < 0.01). Although co-cultivation with *M*. *alternatus* yielded 22.8% dauer larvae, the percentage of dauers on beetles was 1.3%, indicating that most dauer larvae were unable to ride on beetles.

## Discussion

We explored the dauer-inducing signals of *B*. *okinawaensis* to clarify the evolution of the dauer-inducing mechanisms and the specific relationship between *B*. *xylophilus* and the cerambycid beetle vectors. However, not only the number of dauer larvae but also the rate of their formation increased over time after nematode inoculation and with increasing nematode population density (Figure 1). In addition, dauer formation was significantly promoted by crude extracts of cultured nematodes, suggesting that water-soluble substances induce their formation (Figure 2). In various nematode species, including free-living and parasitic nematodes (e.g., *C*. *elegans*, *Pristionchus pacificus* Sommer, Carta, Kim, and Sternberg, 1996 (Nematoda: Diplogastridae), *Heterorhabditis bacteriophora* Poinar, 1975 (Nematoda: Heterorhabditidae), and *Parastrongyloides trichosuri* Mackerras, 1959 (Nematoda: Strongyloididae), some water-soluble ascarosides identified or suggested as dauer-inducing pheromones (Golden and Riddle, 1982; Jeong et al., 2005; Butcher et al., 2007; 2008; Bose et al., 2012; Noguez et al., 2012; Stasiuk et al., 2012; Bose et al., 2014). All dauer-inducing ascarosides for other nematode species have not been identified in *B. xylophilus* (Choe et al., 2012). Although *B*. *xylophilus* secretes the ascarosides asc-C5 (ascr#9) and asc-ΔC6 (Zhao et al., 2016; Meng et al., 2020; Zhao et al., 2021), no reports are indicating that these ascarosides have roles to induce dauer formation. We did not identify the active substances in the crude extract of *B*. *okinawaensis.* However, it is likely that some form of ascaroside, as in other nematodes, is involved in the induction of dauer larvae of *B*. *okinawaensis*.

Incubation at high temperature also promoted dauer formation in *B. okinawaensis* (Figure 3). *Caenorhabditis elegans* dauer formation is also enhanced at high temperatures in the presence of pheromones (Golden and Riddle, 1984). In the absence of pheromone, the population of *B. okinawaensis* did not proliferate well at 20°C (Figure 3A) and the number of dauer was extremely low. This result is consistent with previous reports that the growth rate of *B. xylophilus* on fungi is maximized at higher temperatures, around 30°C (Futai, 1980; Pimentel and Ayres, 2018). However, a wide range of environmental stimuli, not only temperature, but also dauer-inducing pheromones, together induce dauer formation in *C. elegans* (Golden and Riddle, 1982; Golden and Riddle, 1984). It is still possible that dauer formation was not promoted at 20°C compared to the other treatments due to the low density of nematodes, resulting in a lower concentration of dauer-inducing pheromones. With the pheromone treatment, about 20% of dauer were induced at 20°C, even though the nematode population was as low as without pheromone, and there was no statistically significant difference in dauer induction rates between at 20°C and 30°C. This suggests that at least lower temperatures do not directly suppress dauer induction, or that dauer induction by higher densities of nematodes dominates at lower temperatures.

When *B*. *okinawaensis* was co-cultivated with pupae of *M*. *alternatus* until the beetles eclosed, dauer larvae were formed at a high rate (Figure 4). In *B*. *xylophilus*, the presence of *M*. *alternatus* is required for the transition from DIII to DIV larvae (Morimoto and Iwasaki, 1973; Maehara and Futai, 1996; Necibi and Linit, 1998; Maehara and Futai, 2001), and DIV larvae are induced by volatile C16 and C18 fatty acid ethyl esters (ethyl palmitate, ethyl linoleate, ethyl oleate, and ethyl stearate), which are more abundant on the body surface of *M*. *alternatus* during adult emergence than in any other stage (Zhao et al., 2013). Although further detailed analysis is required, it is possible that the dauer stage of *B*. *okinawaensis* may be induced by the same or similar compounds on *Monochamus* beetles. Notably, dauers of *B*. *okinawaensis* induced by *M*. *alternatus* in this study could rarely ride on beetles (1.3%), although dauer formation was promoted by *M*. *alternatus* to the same level as *B*. *xylophilus* (Maehara et al., 2020). Indeed, in a previous study, a small percentage of dauers of *B*. *okinawaensis* was vectored by *M*. *alternatus* (0.14%) (Maehara and Kanzaki, 2016). The DIV larvae of the xylophilus group can ride several cerambycid beetles species under laboratory conditions. For example, DIV larvae of *B*. *xylophilus* can transfer to not only the natural vector *M*. *alternatus* (percentage of dauers on beetles to total dauers: 35.8%) but also cerambycid beetles of other genera: *Acalolepta luxuriosa* Bates, 1873 (Coleoptera: Cerambycidae) (22.2%), *P. hilaris* Pascoe, 1857 (Coleoptera: Cerambycidae) (12.5%), and *Acalolepta fraudatrix* Bates, 1873 (Coleoptera: Cerambycidae) (7.7%) (Maehara et al., 2020). Therefore, the transfer ability of *B*. *okinawaensis* to cerambycid beetles was less than that of *B*. *xylophilus* although *M*. *alternatus* is not a native vector for *B*. *okinawaensis*. Indeed, the number of dauer larvae of *B*. *okinawanesis* carried by *M*. *maruokai* in nature is <20 per beetle (Kanzaki et al., 2008). However, this low transmissibility may not negatively impact survival because *B*. *okinawaensis* is a self-fertilizing hermaphrodite; thus, the dispersal of a small number of nematodes to a new environment may be sufficient to maintain the population.

In conclusion, DIII larvae of *B*. *okinawaensis* were induced at high nematode population densities and by the presence of the beetle vector. *Bursaphelenchus xylophilus* DIII and DIV can be induced by an increase in nematode population and the presence of the vector, respectively (Morimoto and Iwasaki, 1973; Maehara and Futai, 1996; Necibi and Linit, 1998; Maehara and Futai, 2001; Zhao et al., 2013; Tanaka et al., 2017). Since only a small percentage of dauers of *B*. *okinawaensis* transferred to *M*. *alternatus*, the DIV larval stage, exclusive to the ‘xylophilus’ group, is probably required for high transfer ability to the beetle vector. DIV larvae of *B*. *xylophilus* are strongly attracted to CO_2_, and enter the tracheal systems of adult beetles, which contain CO_2_ (Miyazaki et al., 1978). This high CO_2_ attraction may facilitate the transfer of DIV larvae of *B*. *xylophilus* to beetle vectors. Among the xylophilus group, which has two distinct stages of dauer, only *B*. *xylophilus* is highly pathogenic to trees, suggesting that its pathogenicity evolved after acquiring the high dispersal ability. This is consistent with the transmission-virulence trade-off model, which suggests that pathogens with high virulence cannot survive without high transmissibility (Ebert and Herre, 1996; Ebert and Bull, 2003). We have developed genomic and genetic tools for *B*. *okinawaensis*, rendering it genetically tractable (Shinya et al., 2014; Sun et al., 2020; Shinya et al., 2022). Investigation of the molecular mechanisms of dauer induction and dauer chemoattraction in *B*. *okinawaensis* and *B*. *xylophilus* will provide insight into the evolution of the trade-off between virulence and transmissibility in *Bursaphelenchus* nematodes.

## Data Availability

The original contributions presented in the study are included in the article/supplementary material, further inquiries can be directed to the corresponding author.
